# Is Kinesio Taping Effective for Sport Performance and Ankle Function of Athletes with Chronic Ankle Instability (CAI)? A Systematic Review and Meta-Analysis

**DOI:** 10.3390/medicina58050620

**Published:** 2022-04-29

**Authors:** Carlo Biz, Pietro Nicoletti, Matteo Tomasin, Nicola Luigi Bragazzi, Giuseppe Di Rubbo, Pietro Ruggieri

**Affiliations:** 1 Orthopedics and Orthopedic Oncology, Department of Surgery, Oncology and Gastroenterology (DiSCOG), University of Padova, 35128 Padova, Italy; pietronicoletti.ft@gmail.com (P.N.); matteo.tomasin.1@studenti.unipd.it (M.T.); giuseppe.dirubbo@studenti.unipd.it (G.D.R.); pietro.ruggieri@unipd.it (P.R.); 2 Department of Neurosciences, Institute of Human Anatomy, University of Padova, 35128 Padova, Italy; 3 Department of Mathematics and Statistics, Laboratory for Industrial and Applied Mathematics (LIAM), York University, Toronto, ON M3J 1P3, Canada; robertobragazzi@gmail.com

**Keywords:** kinesio taping, chronic ankle instability, ankle sprain, ankle injuries, elastic taping

## Abstract

*Background and Objectives:* Ankle injuries are the most common type of injury in healthy active individuals. If not treated properly, recurrent sprains can lead to a condition of chronic ankle instability (CAI). The aim of the present review is to evaluate the effects of Kinesio Taping (or KT) on sports performances and ankle functions in athletes with CAI. *Materials and Methods:* This systematic review with meta-analysis was carried out following the criteria of the Prisma Statement system (registered on Open Science Framework, number: 10.17605/OSF.IO/D8QN5). For the selection of the studies, PubMed, Scopus and Web of Science were used as databases in which the following string was used: (“kinesiology tape” OR “tape” OR “taping” OR “elastic taping” OR “kinesio taping” OR “neuro taping”) AND (unstable OR instability) AND (ankle OR (ankle OR “ankle sprain” OR “injured ankle” OR “ankle injury”)). The Downs and Black Scale was used for the quality analysis. The outcomes considered were gait functions, ROM, muscle activation, postural sway, dynamic balance, lateral landing from a monopodalic drop and agility. Effect sizes (ESs) were synthesised as standardized mean differences between the control and intervention groups. Calculation of the 95% confidence interval (CI) for each ES was conducted according to Hedges and Olkin. *Results:* In total, 1448 articles were identified and 8 studies were included, with a total of 270 athletes. The application of the tape had a significant effect size on gait functions, ROM, muscle activation and postural sway. *Conclusions:* The meta-analysis showed a significant improvement in gait functions (step velocity, step and stride length and reduction in the base of support in dynamics), reduction in the joint ROM in inversion and eversion, decrease in the muscle activation of the long peroneus and decrease in the postural sway in movement in the mid-lateral direction. It is possible to conclude that KT provides a moderate stabilising effect on the ankles of the athletes of most popular contact sports with CAI.

## 1. Introduction

Ankle injuries are the most common injury in healthy active individuals [1,2,3], affecting women more frequently than men (13.6 vs. 6.94 per 1000 exposures), children more frequently than adolescents (2.85 vs. 1.94 per 1000 exposures) and adolescents more frequently than adults (1.94 vs. 0.72 per 1000 exposures) [4]. These high incidence rates show that these injuries can cause high costs for health care systems; Gribble et al. showed that ankle injuries cost USD 6.2 billion in high school athletes in the US and EUR 208 million in the Netherlands annually [5,6].

The sports in which ankle injuries are most common are indoor and court sports [7,8], with an incidence rate of 7 per 1000 exposures, compared to water/ice sports (3.7/1000 exposures), field-based sports (1.0/1000 exposures) and outdoor pursuits sports (0.88/1000 exposures) [4]. About 30% of ankle injuries occur during training sessions and the remaining 70% during matches, where performance becomes much more demanding [9,10,11,12].

Chronic ankle instability (CAI) is the process caused by repetitive ankle sprains and multiple episodes of the ankle “giving way” with persistent symptoms [13,14,15]. It mainly affects the sports population and is related to multiple inversion injuries [16,17]. The prevalence of CAI in a population with a history of ankle injuries is 46%, ranging from 9 to 76% [2]. The wide range in prevalence data is influenced by multiple factors, such as gender and age, which can have a very important impact on the development of this type of injury [18]. As mentioned above, women and young people are more likely to develop ankle injuries and CAI [19,20]. The meta-analysis by Chiao-I Lin et al. showed that the prevalence of CAI was much higher in subjects under 18 years old, with a rate of 63% compared to the entire population considered [2].

In the study by Chiao-I Lin et al., recurrent ankle sprain (61%) was most prevalent in soccer athletes, and the highest rate of perceived ankle instability (41%) was in track and field athletes with a history of ankle sprain [2].

This type of instability can be related not only to mechanical instability or ligamentous laxity but also to functional instability, with frequent “giving way” during normal daily activities [21,22,23]. If the soft tissues are not damaged despite repeated injuries, then the clinical condition is identified as functional ankle instability (FAI) [24]. The risk factors underlying CAI are not exclusively linked to ligament laxity but also to a proprioceptive deficit, to muscle weakness of the lateral compartment of the leg, mainly the peroneus brevis and longus, to their delayed neuromuscular activation and to a loss of static and dynamic balance in a monopodalic load [25,26,27,28,29,30]. Other risk factors are related to high BMI, participation in sports, having an increased talar curvature and not using external supports [31].

To establish the severity of ankle instability in people affected, three scores with a defined cutoff score are recommended by the International Ankle Consortium: The Ankle Instability Instrument (AII), answering “yes” to at least five questions; The Cumberland Ankle Instability Tool (CAIT), with <24 points and The Identification of Functional Ankle Instability (IdFAI), with >11 points [32].

Since CAI can become a demanding issue for athletes who are forced to stop at every episode of sensation of “giving way” of their ankle, it is important to look for prevention strategies and methods that improve the condition and performance of athletes. Given this background and the incidence of these injuries, it was important to evaluate how the kinesio taping (KT) acts on the injured ankle [33]. This elastic bandage was introduced in the 1970s by Kenzo Kase and has become very popular over the last few decades, used widely in physiotherapy for musculoskeletal disorders affecting both the upper and lower limbs. In particular, it has become more widespread in sports and other rehabilitation fields due to its intrinsic stretching capacity, which allows it to maintain sufficient mobility in the areas where it is applied compared to inelastic bandages [34,35]. A very important aspect of KT is its ability to retract after being applied due to its surface texture, which allows a slight traction of the underlying tissues, granting greater stability to the targeted area [36,37].

As KT is widely used for multiple dysfunctions with debated effect reported in the literature, and the several studies published over the years seem to contradict one another, the aim of this review was to investigate the real effectiveness of KT in improving the specific performance and the ankle function of athletes with CAI during sports activities such as soccer, basketball, volleyball, baseball and badminton in which this disorder is frequent and challenging to treat [38,39,40].

## 2. Materials and Methods

### 2.1. Data Sources and Search Strategy

Findings of the present systematic review and meta-analysis are reported according to the “Preferred Reporting Items for Systematic Reviews and Meta-Analysis” (PRISMA) guidelines [41]. The study protocol has been registered within the Open Science Framework depository (Identifier code: DOI:10.17605/OSF.IO/D8QN5).

A search string related to KT was devised based on three major components: kinesiology taping, ankle and instability. For each component, an exhaustive list of keywords and relevant synonyms were generated, using proper Boolean connectors. The following string was used: (“kinesiology tape” OR “tape” OR “taping” OR “elastic taping” OR “kinesio taping” OR “kinesiotape” OR “neuro taping”) AND (unstable OR instability) AND (ankle OR (ankle OR “ankle sprain” OR “injured ankle” OR “ankle injury”)). 

The existing literature was systematically searched from 2010 to December 2021 using the databases PubMed/MEDLINE20, ISI/Web of Science (WoS) and Scopus. A language filter was also applied, searching in English only.

### 2.2. Inclusion Criteria

Only articles written in English and designed as original studies were included. Only randomised clinical trials (either cross-sectional or cross-over), cohort studies, case-control studies and case series were selected that met the following criteria: the participants were adults, both females and males, with a diagnosis of chronic ankle instability (CAI); the participants were athletes; there was at least one intervention group; there was at least one group who had KT applied to their ankle; at least one ankle function was analysed in the groups.

### 2.3. Exclusion Criteria

All studies that included non-athletic patients or those who underwent ankle surgery or had an ankle fracture of at least 6 months were excluded. Non-English-language articles, review articles, meta-analyses, editorials, letters, comments, conference abstracts or case reports, duplicate or non-full-text articles were also excluded.

### 2.4. Screening

This systematic procedure, according to PRISMA guidelines, consists of identification, screening, assessment and inclusion of those studies and the relative patients included that were suitable for the review aims [41]. Hence, the screening was carried out by reading first the abstracts of all of the articles found. If the abstracts met the inclusion criteria, the full-text manuscript was retrieved and assessed. A cross-reference search of the selected articles was also performed to obtain other relevant articles for the study.

After this initial process, the selected articles and references were reviewed and assessed independently by two reviewers (GDR and MT), and all queries were discussed and resolved by the supervisory team (CB and PN) during regular meetings. If there was disagreement among the investigators regarding the inclusion or exclusion criteria, the senior investigator (PR) made the final decision. The level of agreement was high, with kappa statistics ≥ 0.80 [42,43].

### 2.5. Data Collection

Finally, data extraction was completed by an independent assessor (NLB). The studies that were selected as includible were ordered in an Excel file in which the data extraction was completed independently. Data were extracted for the various studies, (authors, publication date, study design, level of evidence, outcome measurements) and for the patients included: numbers, sex, age, type of sport.

### 2.6. Quality Appraisal

The quality analysis was carried out using the Downs and Black Scale [44], attributing to each item 1 point if the study fully complied with the criteria and if not, 0 points. The only exception was made for item 5 of the scale where 1 point was awarded even if the criteria was not fully met or was partially met, and 2 points instead if the criteria was fully met. The average value was calculated among the total scores to define the average quality of the articles included in the systematic review and meta-analysis.

### 2.7. Meta-Analysis

Effect sizes (ESs) were synthesised as standardized mean differences between the control and intervention groups, correcting for the small sample size when necessary (Hedges’ g). Calculation of the 95% confidence interval (CI) for each ES was conducted according to Hedges and Olkin (1985) [45]. For studies with a pre- and post-design, overall ES was computed both sensu Morris (2002) and sensu Klauer (2001) [46]. While the latter computes the overall ES simply subtracting the pre-ES from the post-ES, Morris (2002) weighs the pre–post mean differences using the pooled pre-test standard deviation [46]. Using Cohen’s rule of thumb, the magnitude and meaning of the computed ES was interpreted as follows: small = 0.20, moderate = 0.50 and large ≥ 0.80 [47]. Fixed-effect models were applied when heterogeneity among studies was not significant according to the I2 statistics; otherwise, a mixed-effect model was applied. All statistical analyses were conducted by means of the commercial software “Statistical Package for Social Sciences” (SPSS version 28.00, IBM Corporation, Armonk, NY, USA). Graphs were generated by means of the commercial software MedCalc^®^ version 20.011 (MedCalc Software Ltd., Ostend, Belgium).

## 3. Results

### 3.1. Search Yield

**Figure 1:**
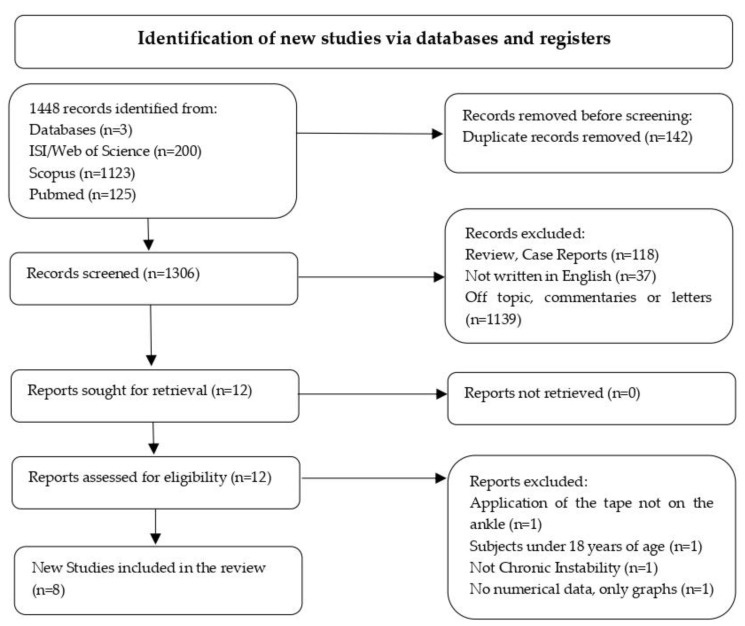
Systematic Reviews and Meta-Analyses (PRISMA) flow chart showing the process for inclusion of papers. For this study, 14 articles were assessed for eligibility after screening: among these, 8 new studies were included in the analysis [41].

The literature search yielded a pool of 1448 items: 1123 from Scopus, 200 from ISI/Web of Science and 125 from PubMed, as shown pictorially in Figure 1. Included studies are reported in Table 1.

### 3.2. Study Characteristics

A total of 270 athletes were included in the review: 171 were male (63.33%) and 99 female (36.66%). The sports of the various groups of athletes were football, basketball, volleyball, baseball and badminton, and college athletes were also present (81 in total). More details of study characteristics are reported in Table 1.

### 3.3. Outcome Measurements

The outcome measurements included were as follows: gait functions including stride velocity, step length, stride length and Heel-Heel (H-H) distance of base of support, measured by the GAITRite Portable Walkaway SystemC; agility through different tests, such as the Illinois Test, 5-0-5, Shuttle Test, Compass Drill Test, T-Agility Test and Figure of 8; dynamic balance by the SEBT and the Y Balance Test; joint ROM; electromyographic muscle activation; lateral landing from a monopodalic drop with the Kistler Force Plate. All of the outcome measurements mentioned above are reported in Table 2 with the tests used to assess them.

### 3.4. Quality Assessment

For the quality appraisal, the Downs and Black scale was used; the studies achieved an average score of 19.25/28, with values ranging from 16 to 22. All items are shown in Table 3.

### 3.5. Meta-Analysis

The effect of KT on dynamic balance was expressed in terms of SEBT. Pooled ES was 0.20 for SEBT (ranging from 0.08 for SEBT-AL to 0.29 for SEBT-L), indicating no significant impact of KT on dynamic balance. Significant large effects could be found for the following: lateral landing in loading time with an ES of 0.717 and a *p*-value of 0.050; gait functions with ES ranging from 1.92 for H-H base support to 2.28 for stride and a *p*-value of 0.000; ROM, only in ankle inversion–eversion angle peak, with an ES of 0.52 and a *p*-value of 0.048; sway parameters, with a relevant ES for sway velocity in medio-lateral direction, with a *p*-value of 1.25; ES for average muscle activity, peroneus longus contraction, with an ES of 0.55 and a *p*-value of 0.042. Further details are reported in Table 4.

## 4. Discussion

CAI is a frequent complication of ankle sprains that may be associated with long-term consequences in athletes. Although taping is a common intervention that is widely used by clinicians and athletic trainers for the treatment of sports injuries and various neuro-musculoskeletal disorders, no studies have evaluated its effectiveness specifically for sports performance and ankle function in athletes affected by chronic ankle instability. 

This is the first systematic review and meta-analysis to investigate only the effect of KT on the sports performances and ankle functions of athletes with CAI. In all of the studies included, KT was analysed as the only treatment implemented on athletes, without concomitant physiotherapy or other types of exercises, so that the potential improvement parameters registered were exclusively attributed to KT. Nevertheless, the recent literature supports a multifactorial approach as the most effective on CAI using multiple interventions such as KT associated with specific proprioceptive exercises [56].

Among the most popular contact sports (football, basketball, volleyball, baseball), the ankle is the joint district most prone to injury [1,2,3]. Without recovering sufficient stability of the ankle, athletes can suffer multiple sprains and relapses during sports seasons, potentially reaching a condition of chronic instability [57,58,59,60]. 

Many articles have been published in the literature about the application of KT in athletes [2,18,19], most of them concerning the upper limb and generally the shoulder complex. In contrast, the available articles about KT and CAI have been very limited and quite recent [48]. This can be explained by the increasing use of KT in recent years and the large interest in evaluating its real effectiveness, even though it is an elastic bandaging technique that was proposed in the early seventies.

Among the eight articles included in this review, the sports performance and ankle functions that could be meta-analysed were (1) gait functions, (2) joint ROM, (3) muscle activation, (4) sway parameters, (5) dynamic balance, (6) lateral landing from a monopodalic drop and (7) agility. The main finding of this review, as reported in Table 4, is that KT had a significant impact only on the following outcomes: (1) gait functions, as reported by Kim et al. [48], who included gait velocity, step length, stride length and Heel-Heel (H-H) distance of the base of support; (2) reducing ankle joint ROM in inversion–eversion; (3) decreasing muscle activation of the peroneus longus; (4) decreasing postural sway in mid-lateral movements, as reported by Sarvestan et al. (2020 [55]).

### 4.1. Gait Functions

In patients with CAI, the entire gait cycle can be altered by an increase in ankle inversion, which can cause both a shorter step length and an increase in the base of support and a reduction in gait speed [61,62]. In our review, the gait functions on which the tape had the greatest impact were the increase in step length and stride length with a relative ES of 2.27 and 2.28, respectively, an increase in speed, with an ES of 1.98 and the reduction in H-H base distance, with an ES of 1.92.

The increase in stride velocity, expressed in m/s, corresponds to a greater looseness during the phases of gait, which, associated with a smaller width of the base of support in dynamics, indicates a greater sense of stability of the athlete during movement [63,64,65,66]. A wider base of support in dynamics usually allows lowering the centre of mass (COM), increasing the body’s stability [67,68].

In the 22 athletes included in the study, the width of the base of support decreased because taping seems to have provided greater stabilisation during walking. The main problem with the study by Kim et al. [48] is the wide range of the confidence interval (95% C.I.) of the gait functions, with values between 1.21 and 3.33; these can be justified by the low number of athletes included in the study, i.e., a very limited sample size, although the methodology of the study was of good quality (21/28).

### 4.2. Ankle Joint ROM

Ankle joint motion has also been found to influence the lower extremity landing pattern in people with CAI [69]. It has been repeatedly confirmed to have a great influence on bilateral postural stability [69,70]. For joint ROM, the only parameter in which taping had a significant impact was in the post-tape reduction in inversion–eversion ankle range, as shown by Sarvestan et al. [55], with an ES of 0.52 and a *p*-value of 0.05, while no substantial change was found in all other joint parameters of the ankle, knee and hip. During the agility tests evaluated by Sarvestan et al. in a previously included study [52], the change in grades in dorsi–plantar flexion during movement was assessed. The results were not included in the meta-analysis because they were not comparable, although Sarverstan et al. reported an improvement in ankle sagittal ROM during linear sprinting. An increase in ankle ROM could reduce the vertical ground reaction forces and the impact on the entire lower limb [30,71]. 

Sarvestan et al. measured the peak joint movement in dorsiflexion and plantar flexion in the sagittal plane and in inversion–eversion in the frontal pllane [55]. It was shown that, after the use of the tape, the joint peak in the frontal plane decreased drastically, limiting excessive rotation of the calcaneus and consequently reducing the oscillations in inversion and eversion during walking, favouring greater stability. Similar results regarding gait functions were also found by Kim et al. [48]. 

Inversion–eversion tilt is a movement that, both in a mechanical and perceptive sense, reduces the feeling of ankle stability [72]. According to Smith et al. [72], application of the tape decreased the sensation of instability in inversion–eversion, suggesting an effect that contributes to preventing recurrent ankle sprains.

### 4.3. Muscle Contraction

In the literature, the possible action of KT in improving muscle contraction is much debated. Some authors speculate that cutaneous stimulation of the tape may induce a greater sensitisation of type 2 mechanoreceptors and improve the recruitment of motor units [73,74]. Other possible explanations may be related to a concentric traction that the tape exerts on the fascia, which may improve muscle contraction by shortening the distance between the origin and insertion of the muscle [75,76,77]. 

In contrast, Sarvestan et al. [55] analysed whether the tape could modify muscle contractions using an electromyographic examination, and an opposite effect was found after application of the KT on the lateral leg muscles. The only muscle among those considered on which the KT had a considerable impact was the peroneus longus with an ES of 0.55 and a *p*-value of 0.05. In the leg with the KT applied, there was a strong decrease in muscle activation justified by a supporting action that the tape provided when applied laterally along the ankle, partially reducing activity of the eversion muscles, especially the peroneus longus. However, this element has both a positive aspect in a phase in which the athlete is looking for an external element of support that allows him to have a more stable ankle during sport performance, but can also have a negative effect on active stabilisation from lateral muscles, which risk being partially lacking and inhibited with the tape on, as demonstrated in Sarvestan et al. [55].

### 4.4. Postural Sway during Movement

Athletes with CAI often do not have instant and corrective ankle reactions when they make contact with the ground. A lack of corrections during movements greatly accentuate body postural sway [72]. Some studies conclude that postural sway depends on a loss of balance, which is an important indicator of possible falls during dynamic performances and in pre-fatigue conditions [78,79,80].

Sarvestan et al. [55] showed that in the mid-lateral direction, KT significantly reduced sway speed and reduced peak acceleration with an ES of 1.25 and a *p*-value of 0.03. In contrast, there were no significant changes in speed and sway area in the anterior–posterior direction. Many studies in the literature have confirmed the effectiveness of KT in improving postural sway parameters, both in relation to speed and sway area, especially in mid-lateral directions [81,82]. Reducing sway velocity in the mid-lateral direction suggests better control in prone-supination movement, corresponding to greater overall stability [82]. 

### 4.5. Dynamic Balance

The meta-analysis for dynamic balance was carried out on the studies of Souza et al. and Gehrke et al. [50,51], which had in common the use of the SEBT test. Data relating to dynamic balance of Alawna et al. [53], in which the Y Balance Test was used, were not meta-analysed, as they were not comparable. Table 4 shows that the ES did not reveal a statistically significant impact, with *p*-values between 0.26 and 0.75. However, both studies only evaluated 34 athletes in total. In the general population with CAI, Hadadi et al. [83] showed that KT had a significant effect on both static and dynamic balance. Other researchers, however, found no improvement in dynamic balance after the application of KT [84,85].

### 4.6. Lateral Landing from Monopodalic Drop

Lateral landing is very difficult, having an impact on the entire lower limb due to the dissipation of energy that is required [86]. For this reason, an alteration in the motor patterns or in the joints involved, such as CAI, can adversely affect the ability to land, even more so if the landing is performed after a monopodalic drop, which is a more challenging function [87,88].

In this review, lateral landing from a monopodalic drop was only assessed by Lin et al. [54] who considered ground reaction forces, loading rate and loading time. The *p*-values of the loading rate and the loading time were between 0.15 and 0.63. Therefore, it was not possible to define a significant impact of the KT on these functions. For the ground reaction forces, although the *p*-values were <0.05 and therefore statistically significant, they had an overall ES—including both the measurements before and after the application of the tape—that did not show a real effectiveness in improving the performance of lateral landing from a monopodalic drop. The values on the CoP (centre of pressure) were not considered for the purposes of the meta-analysis because they did not include interquartile ranges, only median values.

In another study [71], Lin et al. also concluded that KT was not sufficient to improve both frontal and sagittal postural control during landing, while Mason-Mackay et al. [89] added that KT must be combined with specific training to improve landing techniques and strategies.

### 4.7. Agility

Agility is an athletic condition that is essential in sports such as those included in this review (football, basketball, volleyball, baseball). This skill allows players to make heterogeneous movements in rapid succession, such as changing direction, turning quickly and cutting, all activities that have a significant impact on the ankle [49,51]. 

In our analysis, agility was assessed by Sarvestan et al. [49] and Gehrke et al. [51] by measuring the time used to perform on tests such as Illinois, 5-0-5, 10-m Shuttle and Figure of 8. No significant improvement was shown, with ES values ranging between −0.35 and 0.34 for males and between −0.53 and 0.31 for females.

### 4.8. Time of Application

In all of the included studies, only Sarvestan et al. [55] reported the time of application of the KT before tests and measurements were carried out. They waited 25 min between application of the tape and the start of the tests.

Some authors have found a positive effect of KT to increase balance and proprioception in patients with CAI between 48 and 72 h [90]. Assessing the effectiveness of KT in the tests seen in this review with a longer application time could be an important aspect to evaluate in future studies.

### 4.9. Limitations and Strengths

There are several limitations in this review. First, since the studies included in the meta-analysis did not evaluate identical outcome variables, there is potential for bias in the validity of our results. However, if multiple data not representing the same outcome of one study were included in the meta-analysis, the weight of that study would increase in proportion. As a result, the total effect would not deviate towards the study with more outcome data. Second, there is no specific technique for KT application: it usually varies according to the symptoms of the patient, the therapist’s experience and intended purpose. This heterogeneity, such as different tensions of the tape or applying KT in different directions and shapes, may have caused inconsistent results and led to non-significant total effects. Furthermore, the taping technique seems to have been kept consistent across the participants in the different studies during activity; however, it was not possible to know how long the taping was kept on during sports performances and the time from taping and injury; this may be affected by variation in the injury mechanisms of the sports. Therefore, it remains to be investigated whether a different taping technique could achieve a better outcome for a specific sport or injury. Finally, KT is not typically used as a single treatment tool but is combined with other treatments such as physical therapy and exercise therapy, aspects influencing the final outcomes that were not possible to evaluate separately. 

Our literature review also has strengths. First, it is the first meta-analysis of randomised controlled trials (7/8) that focuses specifically on the effectiveness of KT. In all studies, except in Sarvestan et al. [55], participants were randomised, which guarantees a more accurate methodology. The eight studies included reported a high level of quality and scientific evidence: an average score of 19.25/28, according to the Downs and Black Scale, and a level of evidence between I and II (three studies I; four level II). Second, the studies included in this review were published recently and very close to each other (from 2017 to 2020), including different sports population groups to guide clinical judgements. Hence, our meta-analysis represents a synthesis of the most current knowledge on this subject in the literature since the seventies. Third, even though this study included ankle function and athletes’ functional performance testing as outcomes to provide more evidence for clinical practice, it was designed with a different approach with respect to previous meta-analyses that reported non-significant results for the effectiveness of KT. 

Finally, since the sports performances and ankle functions analysed among the included studies were heterogeneous, several independent meta-analyses were carried out, stratifying the outcomes according to subgroups to avoid comparing outcomes considering different parameters. There may be disparate ankle functions and performances that require very specific skills and ad hoc evaluation tests, especially when comparing different sports and related parameters. Through the meta-analysis of each outcome, it was possible to evaluate the efficacy of KT for every single item, avoiding overall conclusions that did not give significance to single parameters for which KT was effective, without omitting any of them. This should be considered as a strength of our study since it ensures the quality, consistency and generalisability of our findings. Furthermore, seven out of eight studies had a sample size of fewer than 35 athletes, which would have increased the risk of bias in a large-scale comparative analysis with heterogeneous data.

Certainly, future studies maintaining the same level of quality of those included in this review but increasing the number of participants and standardized outcome measurements are required to better clarify the role of KT in CAI. In addition, further investigation is necessary to define the influence of psychological aspects on the athletes’ performances during KT application [9,10,11,71]. It is possible that individuals with CAI perceive their ankle to be more stable and have more confidence in their ankle and their ability to perform challenging tasks because of factors such as increased mechanical stability or a placebo effect of the tape [91,92,93]. Although not sufficient, there is considerable evidence in the current literature for the psychological effects of taping.

## 5. Conclusions

The present systematic review and meta-analysis shows that KT, used on athletes with CAI (playing football, basketball, volleyball, baseball and badminton), is effective only on some of the performances and ankle functions analysed. It was not possible to define the precise time of the application of KT to the ankle joint of the athletes included to see the benefit in performing sports. However, the meta-analysis showed a significant improvement particularly on the following: gait functions (step velocity, step and stride length and reduction in the base of support in dynamics); reduction in the joint ROM in inversion–eversion; decrease in the muscle activation of the long peroneus; decrease in the postural sway in movement in the mid-lateral direction. 

In contrast, other aspects such as dynamic balance, lateral landing from a monopodalic jump and agility tests did not improve significantly by applying KT to the ankle joint. 

Finally, as the improvement achieved by some of the parameters analysed reflects an increased stabilisation of the ankle joint of these athletes during sports performance, it is possible to conclude that KT has a moderate stabilising effect on the ankles of the athletes of the most popular contact sports with CAI.

## Figures and Tables

**Table 1:** Study Characteristics.

Author (Publication Year)	Type of Study	Level of Evidence	*n* (m/f)	Age (Years *)	Sport
Kim et al. (2017) [48]	Cross-Over Randomised Design	I	22 (m)	17.72 ± 0.76	Football
Sarvestan et al. (2018) [49]	Cross-Sectional Randomised Design	II	26 (13 m/13 f)	23.9 ± 1.6	University Athletes
Souza et al. (2018) [50]	Cross-Sectional Randomised Trial	II	13 (9 m/4 f)	23.2 ± 3.2	Basketball
Gehrke et al. (2018) [51]	Cross-Sectional Randomised Trial	II	21 (14 m/7 f)	23.7 ± 3.2	Basketball
Sarvestan et al. (2019) [52]	Cross-Sectional Randomised Design	II	25 (13 m/12 f)	23.8 ± 1.62	College Athletes
Alawna et al. (2020) [53]	Randomised Controlled Trial	I	100 (56 m/44 f)	22.25 ± 2.96	Volleyball
Lin et al. (2020) [54]	Randomised Controlled Trial	I	33 (25 m/8 f)	22.0 ± 2.8	Basketball, volleyball, baseball and badminton
Sarvestan et al. (2020) [55]	Case-Control Study	III	30 (19 m/11 f)	23.91 ± 2.58	College Athletes
**TOTAL**			270 (171 m/99 f)		

* age = mean ± SD.

**Table 2:** Outcome measurements.

Author (Publication Year)	*n* (m/f)	Outcome Measurements	Test
Kim et al. (2017) [48]	22 (m)	Gait Functions	GAITRite PORTABLE WALKAWAY SYSTEMc (cm)
Sarvestan et al. (2018) [49]	26 (13 m/13 f)	Agility	Illinois, 5-0-5, 10-m Shuttle, Hexagon, Compass Drill, T-Agility Test (*s)
Souza et al. (2018) [50]	13 (9 m/4 f)	Dynamic Balance	SEBT (*cm)
Gehrke et al. (2018) [51]	21 (14 m/7 f)	Dynamic Balance Agility	SEBT (cm) Figure-of-8 (s)
Sarvestan et al. (2019) [52]	25 (13 m/12 f)	ROM during Agility tests	Illinois, 5-0-5, 10-m Shuttle, Hexagon, Compass Drill, T-Agility Test (s)
Alawna et al. (2020) [53]	100 (56 m/44 f)	Dynamic Balance *ROM Vertical Jump	Y Balance Test (inches) ROM (degrees) Vertical Jump (inches)
Lin et al. (2020) [54]	33 (25 m/8 f)	Lateral landing performance in single-leg drop	KISTLER FORCE PLATE PEAK *vGRF (%BW), Loading Rate (N/ms), Loading Time (ms), Difference of *CoP-range, Difference of CoP-velocity
Sarvestan et al. (2020) [55]	30 (19 m/11 f)	Postural sway parameters ROM Muscle Activation	KISTLER FORCE PLATE PEAK (cm) ROM (degrees) EMG (% peak)
**TOTAL**	270 (171 m/99 f)		

*s = seconds, *cm = centimetres, *ROM = range of movement, *vGRF = ground reaction forces, *CoP = centre of pressure.

**Table 3:** Quality Assessment with Downs and Black Scale.

ITEM	Kim et al. (2017) [48]	Sarvestan et al. (2018) [49]	Souza et al. (2018) [50]	Gehrke et al. (2018) [51]	Sarvestan et al. (2019) [52]	Alawna et a.l (2020) [53]	Lin et al. (2020) [54]	Sarvestan et al. (2020) [55]
1.	1	1	1	1	1	1	1	1
2.	1	1	1	1	1	1	1	1
3.	1	1	1	1	1	1	1	1
4.	1	1	1	1	1	1	1	1
5.	2	1	2	2	0	2	2	0
6.	1	1	1	1	0	1	1	1
7.	1	1	1	1	1	1	1	1
8.	0	0	0	0	0	1	1	1
9.	0	0	0	0	0	0	0	0
10.	0	0	1	1	1	0	0	1
11.	1	1	1	1	1	1	1	1
12.	1	1	1	1	1	1	1	1
13.	1	1	1	1	1	1	1	1
14.	1	0	1	0	0	1	0	0
15.	1	0	1	1	0	0	0	0
16.	0	0	0	0	0	0	0	0
17.	1	1	1	1	1	1	1	1
18.	1	1	1	1	1	1	1	1
19.	1	1	1	1	1	1	1	1
20.	1	1	1	1	1	1	1	1
21.	1	1	1	1	1	1	1	1
22.	U/D	U/D	U/D	U/D	U/D	U/D	U/D	U/D
23.	1	0	1	1	0	1	1	0
24.	1	0	1	1	0	1	1	0
25.	1	1	1	1	1	1	1	0
26.	0	0	0	0	0	0	0	0
27.	0	1	0	0	1	0	0	0
**TOTAL**	21/28	17/28	22/28	21/28	16/28	21/28	20/28	16/28

U/D = undetermined.

**Table 4:** Results.

Parameter	Effect Size or ES (SMD)	Standard Error	95% CI	*p*-Value	*I^2^*
Dynamic Balance					
SEBT	0.197	0.237	−0.268 to 0.662	0.406	0.00%
SEBT-A	0.0979	0.237	−0.375 to 0.571	0.681	0.00%
SEBT-AM	0.269	0.238	−0.206 to 0.744	0.263	0.00%
SEBT-M	0.199	0.237	−0.275 to 0.673	0.405	0.00%
SEBT-PM	0.211	0.237	−0.263 to 0.685	0.377	0.00%
SEBT-P	0.187	0.237	−0.286 to 0.661	0.433	0.00%
SEBT-PL	0.250	0.238	−0.224 to 0.725	0.296	0.00%
SEBT-L	0.286	0.238	−0.189 to 0.761	0.234	0.00%
SEBT-AL	0.0753	0.237	−0.398 to 0.548	0.752	0.00%
**Lateral Landing**					
Kistler force plate peak vGRF—ground reaction forces	0.09 (overall ES sensu Morris) 0.134 (overall ES sensu Klauer)				
	0.588 (pre)	0.246	0.095 to 1.081	0.017	0.00%
	0.455 (post)	0.249	−0.034 to 0.943	0.068	0.00%
Loading Rate	0.243 (overall ES sensu Morris) 0.233 (overall ES sensu Klauer)				
	0.127 (pre)	0.246	−0.356 to 0.61	0.606	0.00%
	0.360 (post)	0.248	−0.126 to 0.846	0.147	0.00%
Loading Time	0.760 (overall ES sensu Morris) 0.836 (overall ES sensu Klauer)				
	0.119 (pre)	0.246	−0.364 to 0.602	0.629	0.00%
	0.717 (post)	0.366	−0.22 to 1.215	0.050	0.00%
**Gait Functions**					
Velocity	1.978	0.368	1.257 to 2.699	0.000	0.00%
Step	2.271	0.387	1.513 to 3.029	0.000	0.00%
Stride	2.277	0.387	1.519 to 3.036	0.000	0.00%
H-H Base support	1.920	0.365	1.205 to 2.634	0.000	0.00%
**Agility**					
Illinois	Male: 0.213 (overall ES sensu Morris)	0.410	−0.59 to 1.02	0.603	0.00%
	0.254 (overall sensu Klauer);	0.410	−0.55 to 1.06	0.536	0.00%
	Female: −0.136 (overall ES sensu Morris)	0.409	−0.94 to 0.67	0.739	0.00%
	−0.186 (overall sensu Klauer)	0.409	−0.99 to 0.62	0.649	0.00%
5-0-5	Male: −0.329 (overall ES sensu Morris)	0.411	−1.14 to 0.48	0.424	0.00%
	−0.425 (overall sensu Klauer);	0.413	−1.23 to 0.38	0.304	0.00%
	Female: −0.412 (overall ES sensu Morris)	0.413	−1.22 to 0.40	0.318	0.00%
	−0.481 (overall sensu Klauer)	0.415	−1.29 to 0.33	0.246	0.00%
10-m Shuttle	Male: −0.351 (overall ES sensu Morris)	0.412	−1.16 to 0.46	0.394	0.00%
	−0.525 (overall sensu Klauer);	0.416	−1.34 to 0.29	0.207	0.00%
	Female: −0.56 (overall ES sensu Morris)	0.417	−1.38 to 0.26	0.179	0.00%
	−0.456 (overall sensu Klauer)	0.414	−1.27 to 0.36	0.271	0.00%
Hexagon	Male: 0.127 (overall ES sensu Morris)	0.409	−0.67 to 0.93	0.756	0.00%
	0.253 (overall sensu Klauer);	0.410	−0.55 to 1.06	0.537	0.00%
	Female: 0.312 (overall ES sensu Morris)	0.411	−0.49 to 1.12	0.448	0.00%
	0.252 (overall sensu Klauer)	0.410	−0.55 to 1.06	0.539	0.00%
Compass Drill	Male: −0.055 (overall ES sensu Morris)	0.408	−0.86 to 0.75	0.893	0.00%
	−0.061 (overall sensu Klauer);	0.408	−0.86 to 0.74	0.881	0.00%
	Female: −0.067 (overall ES sensu Morris)	0.408	−0.87 to 0.73	0.870	0.00%
	−0.092 (overall sensu Klauer)	0.408	−0.89 to 0.71	0.822	0.00%
T-Agility Test	Male: 0.339 (overall ES sensu Morris)	0.411	−0.47 to 1.15	0.410	0.00%
	0.341 (overall sensu Klauer);	0.411	−0.47 to 1.15	0.407	0.00%
	Female: −0.402 (overall ES sensu Morris)	0.413	−1.21 to 0.41	0.330	0.00%
	−0.415 (overall sensu Klauer)	0.413	−1.22 to 0.39	0.315	0.00%
Figure of 8	0.302	0.310	−0.307 to 0.910	0.331	0.00%
**ROM**					
Ankle angle peak Dorsi–-Plantar flexion	0.03	0.258	−0.48 to 0.54	0.908	0.00%
Ankle angle Inversion–Eversion	0.52	0.263	0.00 to 1.04	0.048	0.00%
Knee angle peak Flexion–Extension	0.01	0.258	−0.50 to 0.52	0.978	0.00%
Hip angle Peak Flexion–Extension	0.05	0.258	−0.46 to 0.56	0.831	0.00%
Hip angle Peak Abduction–Adduction	0.12	0.258	−0.39 to 0.63	0.794	0.00%
**Sway parameters**					
Sway length	0.14	0.259	−0.37 to 0.65	0.436	0.00%
Sway area	0.37	0.261	−0.14 to 0.88	0.499	0.00%
Sway displacement anterior–posterior	0.15	0.259	−0.36 to 0.66	0.433	0.00%
Sway displacement medial–lateral	0.46	0.262	−0.05 to 0.97	0.162	0.00%
Total velocity	0.16	0.259	−0.35 to 0.67	0.436	0.00%
Sway velocity anterior–posterior	0.17	0.259	−0.34 to 0.68	0.433	0.00%
Sway velocity medial–lateral	1.25	0.284	0.69 to 1.81	0.029	0.00%
**Average muscle activity (% Peak)**					
Lateral Gastrocnemius	0.01	0.258	−0.50 to 0.52	0.963	0.00%
Medial Gastrocnemius	0.01	0.258	−0.50 to 0.52	0.901	0.00%
Tibialis Anterior	0.06	0.258	−0.45 to 0.57	0.674	0.00%
Peroneus Longus	0.55	0.263	0.03 to 1.07	0.042	0.00%

## Data Availability

Not applicable.
